# Spatiotemporal Pixelization to Increase the Recognition Score of Characters for Retinal Prostheses

**DOI:** 10.3390/s17102439

**Published:** 2017-10-24

**Authors:** Hyun Seok Kim, Kwang Suk Park

**Affiliations:** 1 Interdisciplinary Program of Bioengineering, Seoul National University, Seoul 03080, Korea; khs0330kr@bmsil.snu.ac.kr; 2 Department of Biomedical Engineering, College of Medicine, Seoul National University, Seoul 03080, Korea

**Keywords:** spatiotemporal, subsampling, character recognition, stimulus frame rates, retinal prosthesis

## Abstract

Most of the retinal prostheses use a head-fixed camera and a video processing unit. Some studies proposed various image processing methods to improve visual perception for patients. However, previous studies only focused on using spatial information. The present study proposes a spatiotemporal pixelization method mimicking fixational eye movements to generate stimulation images for artificial retina arrays by combining spatial and temporal information. Input images were sampled with a resolution that was four times higher than the number of pixel arrays. We subsampled this image and generated four different phosphene images. We then evaluated the recognition scores of characters by sequentially presenting phosphene images with varying pixel array sizes (6 × 6, 8 × 8 and 10 × 10) and stimulus frame rates (10 Hz, 15 Hz, 20 Hz, 30 Hz, and 60 Hz). The proposed method showed the highest recognition score at a stimulus frame rate of approximately 20 Hz. The method also significantly improved the recognition score for complex characters. This method provides a new way to increase practical resolution over restricted spatial resolution by merging the higher resolution image into high-frame time slots.

## 1. Introduction

Several groups have developed visual prostheses to restore the vision of blind people by electrical stimulation of visual pathways using implanted microelectrodes [1,2,3,4,5,6]. As a result, Argus II and alpha-IMS systems are now commercially available [1,2]. The quality of restored vision is dependent on various factors, such as the limited number of microelectrodes and the “interplay between stimulating technology and underlying neurophysiology of the retina” [7,8]. An image-processing algorithm is required to optimize the transmitted image information under the given spatial resolution.

The reading ability is one of the visual tasks that can be restored when the sight of blind people is restored through retinal prosthesis implantation. Many researchers have investigated the psychophysics of pixelized reading using various parameters and methods of finding adequate parameters with the goal of overcoming the problem of limited number of microelectrodes [9,10,11].

Sommerhalder et al. studied the recognition of single letters and words to find the appropriate number of pixels using simulated prosthetic vision [12]. About 300 pixels were needed to recognize close-to-perfect reading with central vision. Sommerhalder et al. continuously studied reading ability using a full-page text-reading experiment [13]. The minimum number of stimulus electrodes required for useful reading ability at eccentric vision was about 600 grid pixels, corresponding to an electrode size of 3 × 2 mm^2^. Fu et al. reported that another important parameter is the window width, which is defined as the number of characters in the field of view, and is inversely proportional to character sampling density [14]. They also showed that the minimum possible resolution of reading ability was 6 × 6 at 15 words/min and 8 × 8 at 30 words/min. Recently, Dagnelie et al. studied the effects of five parameters of pixelized images—dot size, grid size, dot spacing, random dropout percentage and gray-scale resolution—when stimuli were provided in the form of paragraphs through a video headset [10]. They reported that all parameters had an influence on the reading speed and accuracy, and suggested that 3 × 3 mm^2^ prosthesis with 16 × 16 electrodes may allow paragraph reading with 90% accuracy at 20–30 words/min. Chai et al. and Zhao et al. studied optimal pixel numbers to recognize individual Chinese characters and to read Chinese paragraphs [15,16]. They used an image-processing algorithm that specialized in Chinese characters to transform original character images into pixelized character images. However, previous researches have focused on using spatial information and relatively little attention was paid to temporal modulation.

Hafed et al. recently reported that fixational eye movements can enhance visual perception by a clinical study of patients with a subretinal visual implant (Alpha IMS) [17]. Alpha IMS can use natural eye movements because it utilizes internal light-sensitive photodiodes without the need for a camera. Human eyes are continuously moving even during visual fixation. In other words, although the human gaze is fixed on one point, the eyes are still moving minutely. This phenomenon is called fixational eye movement. Human eye movements can be divided into three parts: tremor, drifts and microsaccades. Each of these movements has different frequencies and roles. However, all of them simultaneously occur even during visual fixation. This fixational eye movement is needed because it helps keep the target objects within the range of human vision and prevents sensory adaptation in the visual path by refreshing the retinal image [18]. Conventional static pixelization methods based on retinal implants with a head-fixed camera, such as Argus II, have not reflected these kinds of real human eye movements.

The aim of this study was to propose a new spatiotemporal pixelization method to improve the performance of visual information transmission. The spatiotemporal pixelization method mimics fixational eye movements by combining spatial and temporal information. The experiments were performed to evaluate the character recognition score of the proposed spatiotemporal pixelization method compared with the conventional static pixelization method. The results showed a higher recognition score using the proposed method. We also discovered that an optimal stimulus frame rate exists for which the score of recognition of characters is maximum.

## 2. Materials and Methods

### 2.1. Image-Processing Methods for Character Pixelization

**Figure 1:**
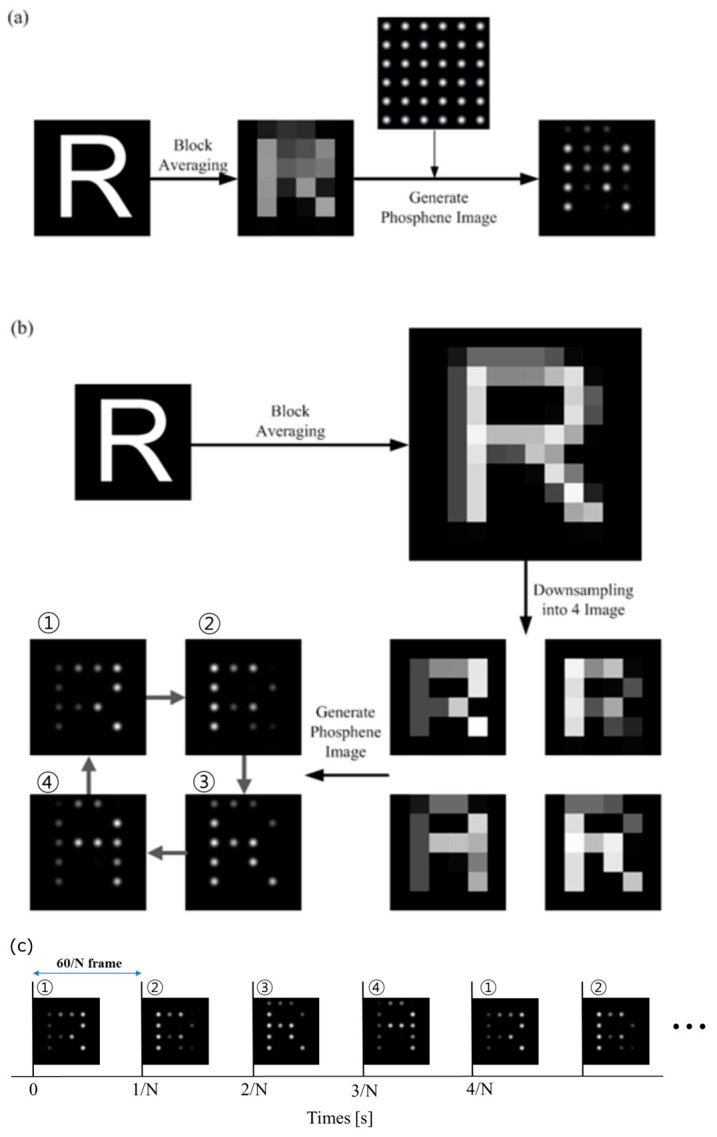
The stimulating image generation procedures. (**a**) The static pixelization method: the image is sampled with the same spatial resolution of the stimulating electrodes for static stimulation; (**b**) The spatiotemporal pixelization method: the original image was sampled at a spatial resolution that was four times higher than that of the static pixelization and sub-sampled into four different lower resolution images; (**c**) The presenting way of the spatiotemporal pixelization method.

To generate pixelized character images for our experiments, we used English letters as well as Korean characters. Prior to beginning the pixelization procedure, we generated input character images for pixelization using commercially available software (Matlab 7.0.1, The Math Works, Inc., Natick, MA, USA). Figure 1 shows stimulating-image-generation procedures for two different pixelization methods. For each image, one English letter or one Korean character in a white Helvetica font was superimposed on a black background and converted to a pixelized image through an algorithm developed using Visual Studio 2005 (Microsoft, Redmond, WA, USA).

- Static pixelization: The original character image was sampled with the same resolution as that of the stimulating array using the block-averaging algorithm, which reduced the resolution of the original image by dividing it into n × n blocks and replacing the pixel values of each block with the mean gray-scale levels of the corresponding block. The phosphene image was generated by convolving the two-dimensional Gaussian function with the block-averaged image; see Figure 1a.
- Spatiotemporal pixelization: By using the block-averaging algorithm described above, the original image was sampled at a spatial resolution that was four times higher than that of the static pixelization. This block-averaged image was subsampled into four different lower resolution images using the following relationship:
  (1) $$I_{ij}\left(x,y\right)=B\left(2x-i,2y-j\right)\;i,j=\left\{0,1\right\}$$
  where $I_{ij}\left(x,y\right)$ is the subsampled image and B(2x−i,2y−j) is the block-averaged image. Four different phosphene images were generated by convolving a two-dimensional Gaussian function to each of the subsampled images. These four phosphene images were seen through the head-mounted display (HMD) with varying stimulus frame rates in the sequence shown in Figure 1b,c. We changed the presented phosphene images at every 1/N second in the abovementioned sequence if the frame rate was N Hz. For example, each phosphene image is presented during six frames if the stimulus frame rate is 10 Hz.

### 2.2. Experimental Designs

All experiments were performed with subjects wearing an HMD (Z800 3DVisor, 800 × 600 resolution, 40° diagonal total field of view, refresh rate 60 Hz, eMagin Co., Bellevue, WA, USA). The pixelized image was shown to the subject through the HMD. Because the HMD substantially reduced the field of view, we reduced the influence of peripheral vision and also made the subjects focus more on the pixelized image during the presentation. Experiments were divided into two sessions based on target character types, English and Korean. Because Korean characters have more complex configurations and require higher spatial resolution for appropriate imaging than English letters, we expected that the character recognition score would be enhanced more significantly for Korean characters using the spatiotemporal pixelization methods.

Before each experimental session, the HMD was fitted to the participant and subjects were told which character type (English or Korean) to expect. The angular substance of the pixelized image was fixed at a visual field of 6.2° × 6.2° on the HMD. The pixelized images of 26 English letters and 40 Korean characters were shown to the subject in random order and displayed for 3 s. Korean characters were selected based on the frequency of their use in words as reported by the National Institute of the Korean Language.

For all subjects, the spatiotemporal method was tested prior to the static pixelization method. Three different image resolutions of images, 6 × 6, 8 × 8 and 10 × 10, were used in both character types. The spatial sizes of the shift were 31.2 arcmin, 23.4 arcmin and 18.6 arcmin, which corresponded to 6 × 6, 8 × 8, and 10 × 10 pixel arrays, respectively. For each session, five different stimulus frame rates (10 Hz, 15 Hz, 20 Hz, 30 Hz, and 60 Hz) were tested for spatiotemporal pixelization to find the optimal stimulating frame rates.

While the image was presented on the HMD, subjects were instructed to read the displayed letter or character loudly and as quickly as possible, and the instructor scored correctness of the answer. Skipping a task, as well as incorrect answers, were regarded as misrecognition. After the experiments, the number of correct answers was divided by the total number of suggested images, and the recognition score was calculated based on the percentage of correct answers. Then, experimental results were compared on two different pixelization methods and as a function of spatiotemporal stimulus frame rates. One-way analysis of variance with post hoc comparisons of the Tukey’s test was used to obtain statistical significance.

Five volunteers who had normal or corrected-to-normal visual acuity participated in this study. Their level of Snellen visual acuity was at least 20/20, and none of them had history of ophthalmic diseases. The subjects were 23–30 years old, and they were all native Korean speakers who also spoke good English and had at least six years of English instruction in schools. Before the experiments, each subject was informed about the experimental procedures as well as the purpose of the study and all of them consented to the experiments.

## 3. Results

### 3.1. Experiment I: Recognition of Pixelized English Letters

**Figure 2:**
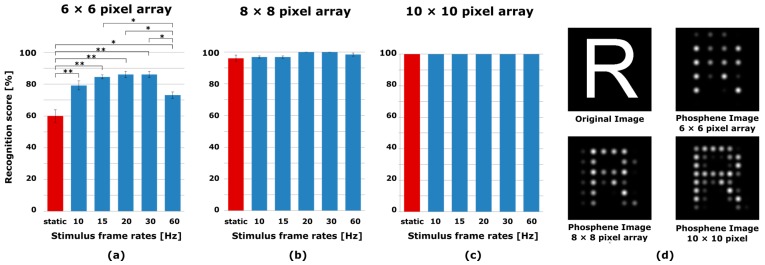
The recognition scores of English letters for 6 × 6, 8 × 8 and 10 × 10 pixel arrays and the example of original and phosphene images. The spatiotemporal pixelization method (blue) was compared with the static pixelization method (red). The result of the recognition score with the (**a**) 6 × 6; (**b**) 8 × 8; and (**c**) 10 × 10 pixel arrays are presented; (**d**) The example of original and phosphene images. Error bars indicate SEM. One-way ANOVA and Tukey post-hoc test, * *p* < 0.05, ** *p* < 0.01.

Five normal subjects were asked to read letters to compare the proposed spatiotemporal pixelization method with the conventional static pixelization method. Figure 2 shows that the recognition score of letters was significantly increased by using the spatiotemporal pixelization method as compared with the conventional static pixelization method in all spatiotemporal stimulus frequencies at the 6 × 6 pixel array (*p* < 0.05). The maximum recognition score was presented at the stimulus frame rate of 20 Hz. However, the recognition scores of 15 Hz, 20 Hz and 30 Hz were only significantly higher than that of 60 Hz. This finding suggests that the stimulus frame rates had affected the recognition process of the visual system. With an 8 × 8 pixel array, we found no significant statistical difference in recognition score between the spatiotemporal and static pixelization methods. With a 10 × 10 pixel array, both methods achieved a recognition score of 100%. No differences between the recognition scores of the two methods were found with an 8 × 8 and 10 × 10 pixel array because the English letters were sufficiently recognizable with the 8 × 8 pixel array.

### 3.2. Experiment II: Recognition of Pixelized Korean Characters

**Figure 3:**
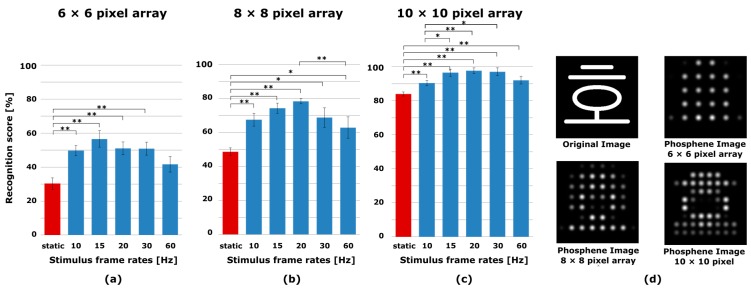
The recognition scores of Korean characters for 6 × 6, 8 × 8 and 10 × 10 pixel arrays and the example of original and phosphene images. The spatiotemporal pixelization method (blue) was compared with the static pixelization method (red). The result of recognition score with (**a**) 6 × 6 pixel array; (**b**) 8 × 8 pixel array and (**c**) 10 × 10 pixel array; (**d**) The example of original and phosphene images. Error bars indicate SEM. One-way ANOVA and Tukey post-hoc test, * *p* < 0.05, ** *p* < 0.01.

The same subjects who participated in experiment I also participated in experiment II, which involved reading pixelized Korean character images. Recognition scores for experiment II are presented in Figure 3. Although the overall recognition score was lower than the score for experiment I due to the increased complexity, the increase in the recognition score of the spatiotemporal method from the static pixelization method was greater than the increase in experiment I. In other words, the recognition score improved much more by employing the spatiotemporal pixelization method in the reading of Korean characters. Because Korean characters have more complex shapes than English letters, the increased recognition score in experiment II suggests that the spatiotemporal pixelization is even more effective for complex images. No statistical differences were observed between the recognition scores of the frame rates with the 6 × 6 pixel array. Meanwhile, the frame rate with 20 Hz scored significantly higher than the frame rate of 60 Hz (*p* < 0.01) with the 8 × 8 pixel array. As regards the 10 × 10 pixel array, the frame rates with 15 Hz, 20 Hz and 30 Hz scored significantly higher than the frame rate with 10 Hz (*p* < 0.05). No significant differences were found between the unexplained frame rates. The stimulus frame rates also affected the recognition score, and the maximum recognition score stayed between 15 Hz and 20 Hz at all three pixel arrays.

## 4. Discussion

### 4.1. Stimulus Frame Rates of the Spatiotemporal Pixelization Method

The most important finding of this study was that the spatiotemporal pixelization method with a specific stimulus frame rate increased the recognition score. The overall results indicate that recognition scores were highest around a specific stimulus frame rate of 20 Hz. When the stimulus frame rate was decreased or increased from 20 Hz, recognition scores decreased in both cases. Low stimulating frame rates seemed insufficient to effectively integrate the four different subsampled images because of the very low presentation cycles, while the high stimulating frame rates seemed to mix four different subsampled images into an almost static image that was difficult to recognize. The recognition scores were presumed to be the highest at an optimal stimulus frame rate that was neither high nor low. We conclude, therefore, that a stimulus frame rate of approximately 20 Hz is optimal for sequentially stimulating four subsampled images to improve the practical recognition resolution under the limitation of microelectrodes pixel numbers.

### 4.2. The Limitations of the Proposed Method

Despite a significant improvement in recognition score demonstrated with the proposed pixelization method compared to the conventional method, there are some limitations to overcome in future studies. First, we experimented with the ideal visual perception case and did not simulate cases of perceptual distortions because of the interplay between the stimulating technology and the underlying neurophysiology of the retina. Second, the performance was evaluated only for single letter images. We have not yet verified the effectiveness of the proposed pixelization method when it is applied to a word or sentence for reading. The simulation also was not performed under real-time reading preparation. Fornos et al. studied reading performance of offline static pixelization compared with real-time static pixelization [19]. They showed that real-time static pixelization leads to better text recognition than offline static pixelization. If individuals can move eyes freely, they can optimize the position of the viewing window for best recognizable pixelized image. However, we believe that applying the spatiotemporal pixelization method to a real-time experiment will also improve the reading performance, because Hafed et al. reported that fixational eye movement can enhance the quality of visual perception [17]. Their scale of microsaccades was approximately 20 arcmin, and the spatial sizes of the shift of the proposed method were less than 32 arcmin within the range of microsaccades, which is similar to that in Hafed et al.’s study. Therefore, the effect of mimicking fixational eye movement can be applied to real-time pixelization based on head-fixed retinal implant systems.

In a future study, we will investigate real-time reading ability using the spatiotemporal pixelization method by adding more subjects, words and sentences to the reading experiments. In addition, we will investigate the effectiveness of our method compared to other visual tasks, such as eye–hand coordination. For example, Li et al. proposed a real-time image-processing method based on global saliency detection and showed significant advantages in eye–hand coordination visual tasks [20].

## 5. Conclusions

We have proposed a new spatiotemporal pixelization method that improves the equivalent spatial resolution in character recognition. The results show improved performance for character recognition compared to the static stimulating method. Recognition scores are also dependent on the stimulus frame rate, which is optimized approximately 20 Hz. By using the proposed spatiotemporal pixelization method, equivalent resolutions of images can be increased over restricted stimulation resolutions of microelectrodes for retinal prostheses.
